# Creating cohesive communities: using Conditional–Collective–Community-Based Incentives to change social norms on polio immunization in Pakistan

**DOI:** 10.3389/fpubh.2025.1575319

**Published:** 2025-06-03

**Authors:** Farhana Tabassum, Zayaan Delawalla, Mushtaq Mirani, Zahid Ali Khan, Aadarsh Fateh Muhammad, Muhammad Asim, Jai K. Das

**Affiliations:** ^1^Institute for Global Health and Development, The Aga Khan University, Karachi, Pakistan; ^2^Department of Society and Technology, AGH University of Krakow, Kraków, Poland; ^3^Community Health Sciences, The Aga Khan University, Karachi, Pakistan; ^4^Department of Paediatrics and Child Health, The Aga Khan University, Karachi, Pakistan

**Keywords:** community engagement, conditional incentives, vaccine hesitancy, polio, social norms theory

## Abstract

Immunization remains a critical public health strategy, particularly in countries like Pakistan where vaccine-preventable diseases are prevalent despite global efforts to eradicate poliomyelitis. This study investigates the role of community engagement (CE) and conditional incentives in increasing polio vaccine uptake in High-Risk Union Councils (HRUCs) of Pakistan. Utilizing an exploratory qualitative research design, the study was conducted to assess the impact of an intervention which involved participatory CE, including the formation of Community Health Committees (CHCs) that conducted community sessions, made home visits, and implemented the Conditional–Collective–Community-Based Incentives (C3Is) in HRUCs in Bannu and Karachi, Pakistan to reduce the rate of refusals for the oral polio vaccine (OPV). These conditional incentives were based on the reduction of polio vaccine refusals by 30 and 50% in specific clusters during the first and second phase of the trial. The findings indicate that leveraging community influencers to change the social norms of the community through CE and C3Is lead to collective behavioral changes. CE served as an effective tool for dispelling myths while also providing a springboard to build community connections and cohesion. Furthermore, this change was accelerated by the provision of conditional communal non-cash incentives, leading to a significant improvement in polio immunization coverage and a reduction in the rate of vaccine refusals. The study underscores the importance of integrating context specific innovative community-specific strategies to overcome vaccine hesitancy and achieve immunization goals in challenging environments and when the target of polio eradication cannot be realized with business as usual.

## Introduction

Immunization serves as a cornerstone of public health interventions worldwide, particularly in countries like Pakistan, where the burden of vaccine-preventable diseases continues to pose significant challenges to child health (1, 2). Over the course of world history, vaccination has played a crucial role in combating various infectious diseases (1). Pakistan embarked on its immunization journey with the initiation of the Expanded Program on Immunization (EPI) in 1978, aimed at providing protection against various diseases, including polio. Pakistan launched its polio eradication program in 1994 with the support of the Global Polio Eradication Initiative (GPEI) (3–5). Subsequent initiatives such as the Stop Transmission of Polio (STOP), sub-national immunization days (SNIDs), and Emergency Operations Center (EOC) have taken significant initiatives in eradicating polio from the country.

**Figure 1:**
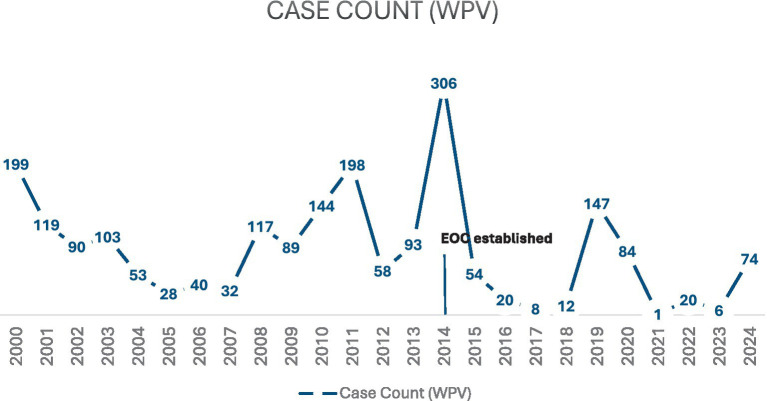
Wild poliovirus cases in Pakistan (9).

Yet the persistence and the recent surge of poliomyelitis cases in Pakistan represents a significant obstacle to completing the global polio eradication initiative (6–8). Currently, Pakistan and Afghanistan are the last two countries in the world that have indigenous poliovirus and are also known as the last two main reservoirs of wild poliovirus (6, 7). Figure 1 highlights the number of cases of wild poliovirus cases in Pakistan since 2000 (9).

**Figure 2:**
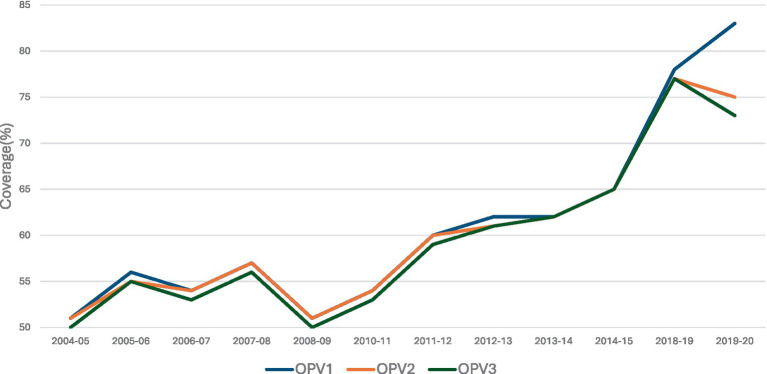
% coverage of OPV 1, 2, and 3 for children aged between 12 and 23 months, in Pakistan (10–18).

There are various factors including misconceptions, fear, vaccine safety, and religious and cultural beliefs regarding the polio vaccine that lead to polio vaccine hesitancy and low vaccine uptake in Pakistan (Figure 2) (10–18), with exposure to messaging about the benefits of polio vaccination not always sufficient (19–22). This is further exacerbated by the flow of migrant and mobile populations crossing the border from Afghanistan into Pakistan and those alternating between their hometowns upcountry and cities such as Karachi (23). Thus, while caregivers’ awareness of the benefits of polio vaccination for their children serves as a strong facilitator of vaccination (24–26), collective community trust plays an important role in the acceptance and vaccine uptake (27). Several studies have highlighted that community members do not always trust the messaging about the vaccine’s effectiveness, importance, or necessity (26, 28, 29). In this context, addressing vaccine hesitancy through tailored communication strategies and CE emerges as an urgent priority (30–33).

CE plays a pivotal role in public health research and program implementation, particularly in low- and middle-income countries (LMICs). CE has shown to have had a significant positive effect on primary immunization outcomes both in terms of coverage and timelines (33–38). However, effective CE requires active involvement and decision making from diverse stakeholders to ensure interventions are contextually relevant and address community concerns (39, 40). To address immunization hesitancy, community engagement and mobilization emerges as a critical tool in Pakistan’s public health repertoire (35, 36, 38). Well-designed and integrated community mobilization campaigns have the potential to fill knowledge gaps, debunk myths, and promote informed decision-making (37, 38, 41, 42). These campaigns leverage communication and social networks to catalyze positive behavior change and bolster vaccine uptake (33, 43).

Despite this understanding of health-related behavioral change, the uptake of current approaches remains low as they rely on health-related behaviors that are frequently difficult to modify and are impacted by a range of structural, social, cultural, cognitive, emotional, and economic factors (44). Thus, introducing any incentive program, such as vouchers, conditional and unconditional cash transfers, and user fee reductions, have been assessed for their impact on child health care seeking, health education participation, and health care visits (45–49). These incentives have also demonstrated the potential to increase coverage of evidence-based interventions by focusing on poverty alleviation and lowering barriers to health care access. The evidence suggests that while CE plays an effective role in enhancing health outcomes, its impact can be boosted by some degree of incentives, as using multiple demand-side interventions in tandem have a greater impact (50). Choosing to implement C3Is stems from the impact conditional community incentives have on health-based outcomes (51–53). Furthermore, this also alleviates problems for communities by providing support on communal non-health outcomes (54).

The success of vaccination programs also hinges on individuals possessing adequate knowledge to make informed decisions regarding vaccine acceptance (43, 55). Thus, it is important to ensure that the dissemination of knowledge creates meaningful behavioral change within individuals. To this end, the social norm theory proposes the idea that individual behavior is shaped by what these individuals perceive as actions that form part of the behavioral norm (56). This theory provides a framework that allows for the exploration of the attitudes, perceptions, and impact peer influence of a community have on an individual. In Pakistan’s public health landscape, CE assumes heightened significance, especially concerning immunization initiatives. The evolution of CE from mere consultation to meaningful partnership underscores the necessity for collaborative efforts in fostering trust and inclusivity (53, 57). Long-term success of such interventions rely on embedding community stakeholders and creating new community-based structures (34, 58). However, if individuals are not adequately engaged through well-designed social mobilization activities, doubts regarding the benefits and risks of vaccination, as well as fears of side effects, persist (42). Successful interventions that enhance knowledge, awareness, and engagement often result in increased coverage of child immunization (43).

To address the significant obstacles hindering vaccination uptake and to boost immunization rates, a pre/post-test quasi-experimental study was conducted (59). The aim was to decrease vaccination refusals and enhance polio immunization coverage through the provision of C3Is coupled with CE. To evaluate the impact of the quasi-experimental study and to gain a more profound understanding of its effects, a qualitative research study was designed, and the results are presented in this paper.

## Theoretical framework

To understand the underlying reasons for vaccination refusals and how to overcome them, the social norms theory provides a framework that consists of descriptive, injunctive, and personal norms as influences on attitudes and perceptions, and consequently the actions and behaviors of individuals within a certain social context. Information and behavior play a crucial role in shaping perceptions, beliefs, and attitudes toward polio immunization in the few remaining endemic locations (60). Externally motivated descriptive norms relate to the individual perceptions of what ‘typical’ behavior is considered in a certain context and relates to their empirical expectations, injunctive norms (also external) are the expectations/perceptions about what ideally should be done (normative expectations), whereas personal norms comprise of the notions of acceptable behavior for the individual themselves, making these internally motivated personal normative beliefs (60–62).

In the context of public health and polio immunization, the social norm theory provides valuable insights into understanding why parents refuse to vaccinate their children. It explores whether this refusal stems from a fundamental distrust of vaccines or from the belief that vaccine refusal is socially acceptable (63). If it is the latter, then individuals may continue to engage in this unhealthy behavior while suppressing their own beliefs and attitudes about what is right. However, it is crucial to analyze the factors that contribute to vaccine refusal using this framework to induce behavior change. Thus, while individuals may personally recognize the benefits of polio vaccines, the descriptive norms within their community may indicate that not everyone chooses to immunize their children against polio. Consequently, individuals may consider the injunctive norms, evaluating how much their community would approve of their decision to reject the vaccine.

This highlights the importance of designing intervention programs centered on C3Is and CE. Bringing about behavioral change requires not only altering individual perceptions of polio immunization but also fostering a community environment where vaccination is socially acceptable and actively desired by all parents. This serves both to align with their perceptions of external behavior and to safeguard their children against the threat of polio.

## Methods

### Study design

A quasi-experimental study was carried out from December 2022 to March 2024 in the Union Councils (UCs) of Districts Bannu and Karachi Central, Pakistan (59). The control clusters were chosen from the same districts, having similar socio-demographics and vaccine refusal rates. This exploratory qualitative study was carried out from January 2024 to May 2024, aiming to assess a participatory CE and demand creation strategy, incorporating trust-building community mobilization and a C3I to reduce vaccine refusals and improve polio immunization coverage. The project activities were structured around the clustering of areas, with each cluster corresponding to the territories overseen by existing polio supervisors.

### Study setting

This study intervention was conducted in two UCs of Karachi and Bannu in Pakistan. These two UCs were selected purposively in consultation with the EOC (59), a multi-sectoral body looking at polio in Pakistan, based on a high polio vaccine refusal rate and hence known as high-risk union councils (HRUCs). A collaboration with EOC was established, both for data-sharing of polio immunization rates to help inform the selection of UCs and for implementing the project in the selected intervention UCs. Meanwhile, the control UCs were selected in discussion with EOC and they were HRUC from the same district with similar refusal rates. HRUCs have been declared by the World Health Organization (WHO) as these UCs have a persistent presence of poliovirus cases coupled with traces of wild poliovirus in the wastewater samples (7). The UC is the lowest administrative unit in the district. The two intervention UCs were Haji Mureed Goth from Karachi and Mir Khel in Bannu, with UC49 in Nazimabad, Karachi and Khwajamad in Bannu as the control groups. The two UCs in Karachi belong to the central Nazimabad district, which has an area of 69km^2^, an approximate population of 3,822,325, and a density of 55,000 people per km^2^ (64). For the UCs selected in Bannu, they were both part of the Bannu district that with an administrative area of 1,972km^2^ and an approximate population of 1,357,890, has a density of 690 people per km^2^ (65). All four of these UCs had been declared as HRUCs by the EOC prior to our intervention and the intervention and control UCs had similar socio-demographic in both study areas.

### Intervention

The intervention UCs was divided into different clusters based on the area supervised by ‘area supervisors’ of the polio program. Based on this, a total of 21 clusters were formed in the two intervention UCs and each cluster had an independent CHC, which was responsible for conducting outreach activities through group sessions and door-to-door visits. To further incentivize the community, C3Is were implemented with a target of 30% reduction in the rate of refusal for vaccines for incentive delivery after 5 months and a 50% reduction after 12 months for the community to be eligible to receive incentives in two tranches (Table 1). CHCs were formed in each cluster comprising locally recognized stakeholders including local politicians, educators, community activists, and religious leaders. Membership of the CHCs was left fluid, allowing for the inclusion of additional stakeholders as the project activities progressed. However, the committees were predominantly male, due to a lack of female mobility in local contexts (Table 2). During the CHC formation phase some women stakeholders were identified and while some continued to engage in the project activities throughout the intervention period, others dropped out at various points due to familial and social refusal. Most of the selected women lived in multistory buildings or compounds, making it convenient for them to visit neighboring families and provide counseling to mothers, thus addressing their fears and concerns.

**Table 1:** An overview of the core project strategies of CE and C3Is.

Role played by CHCs	Establishing channels of communication within their communities.
	Dispelling of myths regarding OPV.
	Demand-creation for the vaccine during polio campaigns.
Leveraging incentives	Dispelling of myths regarding OPV.
	Demand-creation for the vaccine during polio campaigns.

**Table 2:** Challenges faced by the CHCs during the project implementation phase.

Karachi	Bannu	Collective
Lack of community cohesion. Needing to convince the male head of the household. This required a change in timings from our CHCS and modifying communication strategies to ensure the men were also involved in CHC activities. Lack of mobility for women in the community due to cultural constraints. Some of those that were initially on boarded to form a CHC withdrew.	Very tight community cohesion, one refusal leads to the entire community refusing. A culture of guns and violence that led to safety concerns and limited mobilization and trust-building activities. Multiple terrorist attacks during the intervention, severely hindering access and mobility of the project staff. Community refusal to engage in CHC activities without any guaranteed incentives. Instead, community members often demanded some incentives themselves. Needing to convince male head of the household to ensure the household became a vaccine acceptor.	Difficulty finding and onboarding CHC members in our target communities. Increased incidence (multiple rounds a month) of polio campaigns causing frustration within the community. Presence of police not just for the security of polio workers but also to enforce the administering of OPV created distrust and resentment. Flawed counting of refusals during polio campaigns as they did not account for those households that get their children vaccinated through healthcare facilities days before the polio campaigns. Frequent postponement of scheduled polio campaigns.

At the start of the project, clusters were formed within each intervention UC (10 in Bannu and 11 in Karachi), with prominent community members reached out to in each cluster and on-boarded to the CHCs. Training sessions at the beginning of the intervention and throughout the intervention period were conducted for CHC members to enhance their engagement toward demand creation strategies through mobilization sessions. These CHCs conducted monthly awareness sessions, door-to-door visits, and distributed Information, Education and Communication (IEC) materials to sensitize the community about childhood diseases and the importance of polio vaccination, addressing vaccine hesitancy. Special attention was given to families who had previously refused polio vaccination during SNIDs. The CHCs were also informed about the C3Is and its two tranches and given a target of 30 and 50% reduction of refusals for the polio vaccine in their respective clusters. However, the incentive was conditional and was only given to those clusters that achieved the targeted reduction in polio refusals. Those clusters that achieved the given target were awarded conditional incentives for community development related initiatives. The decision to provide C3Is was informed by previous interventions that utilized this novel approach finding that community mobilization alone does not lead to the desired behavioral change (66). Thus, by having community members decided mutually about the utilization of these conditional incentives in their respective communities, it created a sense of ownership and pride within the intervention communities while also improving non-health outcomes. This included the provision of a solar energy system to public sector girls’ schools, procuring sewing machines for women and deep fryer machines and vegetable carts for men to enhance their income generation, facilitating the construction of a community mosque, repairing streets, establishing water supply pipelines to community parks, and providing wheelchairs and tricycles for the differently abled members of the community.

### Participants and sampling

For this qualitative evaluation purposive sampling was utilized for conducting in-depth interviews and focus group discussions. This sampling technique allowed for a representative mix of respondents from our UCs based on gender and views about polio immunization. All the study participants were selected as per predefined eligibility criteria and included firstly key stakeholders within the intervention communities that actively participated and engaged in activities related to the project, secondly, we also interviewed parents with at least one child under the age of 5 in our target communities with the eligibility criteria being those parents who either initially refused the polio vaccine but now accept it as a result of the intervention or parents who attended the group community sessions. The last participant category was the project staff with only those interviewed that were directly involved in the project activities (Table 3).

**Table 3:** Participants included in the study.

Participant categories	Study sites		Eligibility criteria
	Karachi	Bannu	
IDIs			
Key stakeholders	4	4	Actively participated and engaged in activities related to the project objectives within the intervention areas (school principal, teachers, social activists, religious leaders, doctors, businesspeople)
Mothers	2	2	Parents who refused polio vaccination but later agreed after intervention; those who attended community sessions
Fathers	2	2	
Total	08	08	
FGDs			
Mothers	3	3	Parents who refused polio vaccination but later agreed after intervention; those who attended community sessions
Fathers	3	3	
Project staff	1	1	Staff directly involved in field activities, planning, execution, and stakeholder engagement
Total	07	07	

### Interview guide

Three separate semi-structured interview guides were developed for the in-depth interviews (IDIs) and focus group discussions (FGDs) (one each for parents, key stakeholders, and project staff). These guides were formulated after a review of the existing literature combined with the knowledge-creation that took place during the project implementation. The major themes of these guides were to gauge the level of knowledge and information about the importance of childhood immunization, the effectiveness of the intervention and any challenges, the role played by providing incentives to the community, and how to ensure sustainability of the project objectives. The guides served as roadmaps, directing conversations toward key themes and enabling in-depth exploration of subtopics that held particular significance. Through this approach, interviews delved into areas of interest with a nuanced perspective, and these were validated by assessing the initial IDIs and FGDs.

### Data collection

Face-to-face interviews were conducted by trained qualitative researchers using the interview guide to maintain consistency while allowing flexibility for participants to express their views naturally. A total of 16 IDIs and 14 FGDs were conducted and interviews lasted between 35 to 50 min. They were conducted in stakeholders’ workplaces or participants’ homes as this ensured privacy, comfort, and ease of participation. The interviews were recorded through digital voice-recorders after taking the written informed consent. Interviews were conducted in Urdu and Pashto, and the audio recordings were later translated into English. An ongoing analysis of the transcripts was conducted and themes generated, with data collection continuing till there was mutual consensus between the authors (FT and ZD) that data saturation had been achieved (67).

### Data analysis

The qualitative analysis followed the epistemological principle of constructivism, aiming to accurately represent participants’ views. The audio recorded data were translated verbatim into English language by the bilingual translator who had the background in public health related field (67). The transcripts were analyzed using both inductive and deductive thematic analysis to develop the coding framework while identifying commonalities, variations, and underlying structures within our dataset to also be reflected in our analysis (68–70). NVivo 14 software was used to organize, code, and analyze interview transcripts, aiding in systematic exploration of themes and patterns, using a deductive approach during coding. Each theme was individually assessed and aligned with the relevant component of the social norm theory by two coders. Themes were then cross-checked and discussed by FT and ZD to ensure a meaningful interpretation. The discrepancies were resolved by discussing with MA and the themes were finalized after mutual consensus of authors (FT, ZD, and MA). Quality assurance was ensured following Lincoln and Guba’s guidelines (70). These criteria encompassed credibility (trustworthiness), neutrality (confirmability), consistency (dependability), and applicability (transferability).

The findings and analysis of the data were presented using the social norms theory framework and mechanisms for norm compliance (60, 71, 72) to understand the reticence of participants and communities toward the polio immunization vaccine, adding another layer of understanding to impact of the community-focused intervention part of this research study (56).

### Ethical approval

The Ethics Review Committee of the Aga Khan University approved the current study’s protocols [ERC # 2024-7,125-27740]. Verbal and written informed consent were obtained from all study participants prior to data collection. Consent included permission to audio record interviews, use anonymized quotes and the option to decline and/or suspend interviews at any point during the study. Participants were encouraged to ask questions and seek clarification.

## Results

The interviews conducted with stakeholders, parents within our intervention clusters and project staff revolved around the communal changes in perceptions toward polio vaccines, childhood immunization, and the effectiveness and sustainability of the intervention.

### Personal norms (personal normative beliefs)—their own beliefs regarding certain behaviors

These personal norms cover a range of themes (Table 4) including beliefs previously and currently held by parents from Bannu and Karachi around the benefits of polio immunization, the importance of the vaccine, and the intervention project in their communities.

**Table 4:** Social norms leveraged to increase polio vaccine uptake in high-risk areas of Pakistan.

Social norm	Definition	Emergent norms in our communities	Summary findings
Personal norms	One’s own belief about certain behaviors (60)	Stakeholders’ Belief on Polio Immunization Parents’ Belief in Being Receptive to CHCs Parents Understand the Significance of Childhood Immunization Parents Accept Polio Vaccine Because They Believe It Is Important Parents Believe Polio Immunization Has No Value/Is Harmful	There is a reciprocal relationship between the internal and external beliefs which was the primary motivator for stakeholders joining our CHCs and for parents to understand the importance of the vaccine.
Injunctive norms	The perceived attitudes or approval of behaviors by others (62)	Community Disapproval of Polio Immunization Non-Acceptance Voluntary Acceptance of Polio Immunization Parents Become Community Mobilizers	Most parents and stakeholders commended the actions of parents that accept and promote the vaccine while disapproving the behaviors of those that continue to refuse it.
Descriptive norms	The perceptions of others’ engagement in certain behaviors (62)	Community Concerns over Polio Immunization Methods & Issues with Polio Workers Community Rejection of Forceful Behaviors During Polio Immunization Rounds Parents Are Accepting Polio Immunization Parents Remain Reluctant Toward Polio Immunization Male Approval Essential for Child Immunization	Refusing vaccinations was the accepted behavior and caused these areas to be classified as HRUCs.
Mechanisms for norm compliance	These mechanisms broadly explain people’s tendency to comply with social norms (71)	Social identity Power Solving of social dilemmas Rewards and punishments	Mechanisms like social identity, power, and rewards actively shifted community norms, reducing polio vaccine refusals through conditional incentives.

#### Stakeholders’ belief on polio immunization

The key stakeholders that formed the CHCs for this project believed that no child should have to suffer through polio. Their altruism led to them taking up this call to action by the project staff and then spreading the call across their communities in both Karachi and Bannu. Some stakeholders mentioned their desire to continue with these sensitization activities and upkeep of the incentives provided after the project period to ensure a 100% immunization rate is met and sustained in their community.

*“Polio has been eradicated globally except for in Pakistan and Afghanistan. So, personally, for every educated person, for every Pakistani, it should be the wish of everyone that we need to do something towards its eradication. We need to end it” (Doctor, Stakeholder, Karachi).*

*“Polio is a vaccine preventable disease, and we can protect our children from this disease by giving them the polio vaccine” (Teacher, Stakeholder, Bannu).*

#### Parents’ belief in being receptive to CHCs

A key component of this intervention study was the mobilization and integration of key community leaders and influential figures in the sensitization of the overall community in a bid to counter the prevalent myths and reduce polio vaccine refusals. Because there was an existing relationship between these community members and the entire community, not only was there a marked difference of the community attitude for this polio campaign compared to previous efforts, but building trust with community members became easier. This relationship was bolstered by the provision of conditional incentives, which allowed CHC members to further their efforts to households that previously refused to participate.

*“No work can ever be done without involving the people of the area. Having [stakeholder name] involved with your project was good because people are impressed by him and he works hard” (Father, Karachi).*

There was respect bestowed to the CHC members when they visited the homes and community members actively took part in the community sessions that focused on the benefits of polio immunization and the consequences of refusals. These sessions, outreach activities, and incentives delivered by the key stakeholders in tandem with the project team formed the core of the project, with all subsequent changes in beliefs and attitudes predicated on these efforts.

*“Community influencers arranged meetings and home visits and gave us an understanding of the benefits of the polio vaccine and the polio campaigns” (Mother, Bannu).*

#### Parents understand the significance of childhood immunization

On the importance of immunization for children, most respondents from Karachi detailed that there is increased susceptibility to diseases such as measles, typhoid, polio, and several other communicable and non-communicable diseases. This was in stark contrast to having little to no information on scheduled immunization routines and their purpose prior to the intervention. Moreover, the growth and lifestyle of their children are also under threat, as they experience worsened symptoms that then become a burden on the health and well-being of the child and on the finances of the parents if non-immunized children do contract any preventable disease.

*“Vaccines fight against diseases for our children. When our children are healthy, when they enjoy good health, then they have everything (Mother, Karachi).*

One participant noted that having a complete immunization course is a travel requirement, putting those unvaccinated at a further disadvantage.

*“Vaccination records are also important for the future as they make travelling possible for us” (Father, Karachi).*

Some participants outlined their experiences getting COVID-19 and how having gotten a vaccine reduced any potential symptoms of the disease – emanating trust in vaccines.

*“A lot of people, including my family members, did not vaccinate against COVID-19 and we lost one of our relatives to the pandemic. That is when I advised them to get vaccinated to prevent further loss against this disease” (Father, Karachi).*

These responses were also echoed by all participants in Bannu, with few participants also making note of the role of herd immunity when it comes to immunization. They correctly identified how those that are not vaccinated are not protected even if the community collectively practices the immunization schedule for children. Participants also believed that it is important to get children vaccinated as they have weaker immune systems and thus require additional help in fighting against diseases, recognizing the importance of polio vaccines as well.

*“When lots of children get vaccinated, it creates a shield of protection, but the children who do not get vaccinated are a threat to others” (Mother, Bannu).*

#### Parents accept polio vaccine because they believe it is important

Most respondents from Karachi claimed to have now become regular participants in the polio campaigns and some mentioned that they now aim to complete the polio vaccination course for their children till they are 5 years of age because of the sensitization and incentive-fueled demand-creation efforts by their CHCs. Furthermore, this has also led to a decrease in refusals from parents from these communities during scheduled polio campaigns conducted by the polio workers.

*“I used to refuse my children getting the vaccine before but now I fully cooperate with the polio team that visits us, and I let them administer polio drops to my children” (Mother, Bannu).*

Respondents from both cities were also overwhelmingly able to correctly identify the consequences of refusing polio vaccinations. These consequences included paralysis that affected mobility, independence, and lifestyle choices, and in some cases even led to death. Furthermore, there was a recognition that there is no cure for the disease, hence, prevention is the best way to avoid a lifetime of pain and disability. A few participants also noted that there is a significant financial burden placed upon the families of those suffering from poliovirus, underscoring the importance of administering polio drops during the formative years of all children.

*“Vaccinations are important for the betterment of our children and their future. I believe it is the responsibility of every parent to get their child vaccinated” (Father, Karachi).*

#### Parents believe polio immunization has no value/is harmful

Both in Karachi and Bannu, there was a proliferation of false information and misconceptions within the respondents based around the reasons that had given polio a bad reputation in our targeted communities subsequently leading to refusals of the vaccine. Coupled with the on-ground optics of the polio campaigns, there remained some pockets of respondents that continued to refuse the administering of the polio vaccine to their children. These optics played a significant role in two ways. First, for the residents of these localities, polio languishes at the bottom of the problem pile—they are in a constant state of making ends meet. Their priorities for better utilities, services, and infrastructure are not being addressed while a microscopic focus on polio led to resentment and displeasure over the priorities of the government. This was particularly true in Bannu, where there was a widespread blatant refusal to engage in any polio-related activities were it not bundled with other primary healthcare objectives or any other quality-of-life interventions. By the inclusion of conditional incentives, the optics of the intervention became favorable within the communities. The second way in which optics led to refusals was because there was an underlying doubt that because this vaccine is administered for free within the confines of their own homes, there is an ulterior nefarious motive for vaccinating children against polio. Thus, onboarding those that reside within the community became a necessity to ensure successful engagement with the project activities.

*“It is all a misunderstanding regarding the polio vaccine. All of us are Muslims, how can one Muslim make another Muslim give anything wrong or Haram?” (Father, Karachi).*

These myths, primarily propagated by religious clerics that claim this vaccine is *Haram* (harmful or forbidden), included sterilization of kids for respondents from both Bannu and Karachi. These reported beliefs were formed based on information from social media, with the only anecdotal evidence of any harmful effects being their kids falling sick or experiencing diarrhea for a short period after the administration of the vaccine.

*“I was not making my children get the polio drops as a lot of people made me scared that my children would grow up unable to have children” (Mother, Karachi).*

### Injunctive norms (normative expectations)—the perceived attitudes or approval of personal behaviors by others

The injunctive norms of the respondents, especially the key stakeholders, led to the emergence of complimentary themes when concerned with polio immunization and its refusal in their communities (Table 4). Having been championing the project, they strongly favored the behaviors that were conducive to the acceptance of polio immunization while expressing equally stern displeasure over actions that increased refusals.

#### Community disapproval of polio immunization non-acceptance

In interviews with CHCs and parents, there was disappointment for the refusals by certain parents within the community. This also was the basis for the primary motivations by the key stakeholders to align themselves with the project’s CE component. Their disappointment and disapproval stemmed from their altruistic desire to see their community thrive, with the threat of polio becoming a significant impediment to that. Thus, when presented with the opportunity to bring about change in the social norms of the community and be a catalyst for the material upliftment of their neighbors through receiving incentives, they joined the CHCs and led the sensitization efforts to understand the reasons for refusals and to dispel any incorrectly held beliefs and misconceptions that feed into the reasons for not allowing their children to be administered an OPV. Furthermore, one participant also expressed explicit displeasure at the treatment meted out to the polio staff at the hands of the parents, with an implicit disapproval of such behavior found in almost all other interviews.

*“The decision of one household to refuse vaccination has a high influence on community behavior and attitude towards refusal cases because if one household refuses from vaccination all the households feel hesitant to give polio vaccination to their children” (Teacher, Stakeholder, Bannu).*

#### Voluntary acceptance of polio immunization

Key stakeholders that formed the CHCs including the parents who already were in favor of polio immunization, and those parents that changed their personal beliefs after the intervention period, all held a favorable view of the polio campaigns after the intervention. This stems from their personal beliefs that not only is the polio vaccine not harmful, instead it provides benefits by preventing polio virus that can leave their children crippled for life while also providing herd immunity for the entire community.

*“It is important for parents to get their children vaccinated because they will be protected from diseases. I did not understand this at first, but then I and many others gained this understanding because of this project” (Father, Karachi).*

#### Parents become community mobilizers

In some clusters, parents that previously refused the OPV became agents of change during the intervention period. They were not formally a part of the CHCs nor were they asked by the project staff to partake in CE or incentive delivery/upkeep activities, yet they served as promoters for the vaccine in their communities working in tandem with the CHC members to increase acceptance of the vaccine within their neighborhoods. When probed about this behavior, these refusers-turned-promoters of the vaccine highlighted that their myths were dispelled and having realized the importance of administering the polio vaccine, they felt it imperative to adhere to the call to action and actively partake in the project’s activities.

*“Community engagement leveraged peer influence to promote vaccination. When community members saw their friends, family members, and neighbors participating in vaccination campaigns, they followed suit, leading to greater vaccine uptake within the community” (Father, Bannu).*

### Descriptive norms (empirical expectations)—perceptions of others’ engagement in behavior

Understanding the descriptive norms around polio immunization required asking respondents about the actions and beliefs of the community. In their responses, parents and stakeholders from both Karachi and Bannu outlined behaviors that they expected from others and noted their disapproval for when behaviors regarding polio immunization deviated from their personal normative beliefs (Table 4).

#### Community concerns over polio immunization methods and issues with polio workers

Our interviews highlighted that community show displeasure over the hostile behavior of the polio vaccination team, arguing that this is only detrimental to the acceptance of the polio vaccine in those households that refuse the dose through polio campaigns. They outlined certain behaviors such as administering drops in the street without parental consent and taking pictures/videos of those parents that refuse the vaccine as disingenuous actions that make the community as a whole hostile to their presence. Moreover, some respondents lamented the lack of education and awareness given by the visiting polio workers regarding scheduled immunization, claiming that they had no knowledge of the importance of vaccinations prior to the community intervention. Furthermore, there were complaints about the hygiene practices of the workers that made them also doubt the maintenance of the cold chain necessary for the OPV.

*“The community had several issues with polio workers such as their general attitude, the way they would constantly bang on doors, and that they would not maintain the cold chain for the vaccine” (Project Staff, Karachi).*

Another recurring complaint that emerged from the parents (and was backed up by the project staff liaising with the polio workers) was the way in which refusals were counted. Even if parents got their children the polio vaccine from a private doctor a couple days before, if they did not participate in the polio campaign, their household was counted as a refusal despite proof that they actively get their children vaccinated.

*“What I have learnt during this entire study is that the polio staff only counts those children that get vaccinated during their campaigns. All those that get vaccinated from other sources are counted as refusals” (Project Staff, Karachi).*

This glaring pitfall in the recording of polio immunization creates additional hindrances toward acceptance and uptake of OPV while casting doubt on the sense of trust and faith established through CE activities.

*“These people [polio workers], they come again and again to disturb us and ask us to present our children so that they can administer the polio drops” (Mother, Karachi).*

#### Community rejection of forceful behaviors during polio immunization rounds

Regardless of their views on the efficacy of the polio drops, parents and key stakeholders expressed displeasure at the ways in which the routine polio campaigns used to be conducted. The most prominent disapproval came from the presence of police. While some respondents admitted that part of their presence was because of the violent behavior polio workers have faced in the past, they felt that by having uniformed officers grouped together with the polio workers, there existed an underlying coerciveness regardless of the parents’ personal norms about polio immunization.

*“The use of police force made us worrisome. Why are they degrading us rather addressing our concerns?” (Mothers, Bannu).*

They also echoed this sentiment regarding the immunization campaigns conducted within schools. Parents noted that such actions erode their trust in the schooling system as the campaigns happen during school hours without parental consent.

*“They [polio workers] would give polio drops to our children forcefully at schools and we would only find out after it had happened” (Mother, Karachi).*

#### Parents are accepting polio immunization

There was an increase in the acceptance of polio vaccination, especially during the polio campaigns in the community. The bulk of the credit to the uptake of immunization rates was accorded to CHCs and their efforts over the past year to sensitize their community members about the benefits of OPV administered during routine polio immunization. The provision of C3Is at two stages during the project cycle served as a catalyst for improving the optics of polio vaccines within the community and increasing trust in the CHCs. These incentives ranged from infrastructure projects in the community schools and parks to providing some community members with the equipment to set up their own small businesses and wheelchairs for some differently abled children in a few communities.

*“The incentives delivery has had a good impact on the community as now the community members understand that polio is not the only focus as the organizations are also catering to the other needs of the community. After receiving the incentives, the committee and community elders have mutually decided to work on the awareness of child vaccination especially regarding polio” (Father, Bannu).*

*“I was also among those that refused the polio vaccine. But due to the hard work and commitment of community influencers, I along with other refusing parents got our children vaccinated with polio vaccine and other routine immunization. I got my daughter vaccinated with the measles vaccine and when my daughter got infected with measles she recovered very quickly. I think that speaks to the success of the project” (Mother, Bannu).*

#### Parents remain reluctant toward polio immunization

Despite the relative success of the incentive delivery and efforts of the CHCs to increase polio immunization rates, few refusals persisted within their communities. This observation was made by the project staff, parents, and key stakeholders alike, with most expressing dismay over not being able to convince those households. Part of the reason for this resistance is the presence of another organization providing residents in the Bannu clusters with specialized nutritious food (SNF) packets. This health-focused initiative led residents to conflate our project with theirs, compounding the demands for additional support beyond polio immunization. The extent of damage caused by this staunch response from the communities hindered trust-building and solidarity within the community, further weakening the effectiveness of CE activities.

#### Male approval essential for child immunization

A consistent theme across both Karachi and Bannu was that the decision to accept or refuse polio vaccine eventually lay in the hands of the men of the household. While CHCs targeted both mothers and fathers and the benefits of the incentives was seen to be communal, the women in the community often deferred the decision to vaccinate on their husband’s approval, making it crucial to sensitize both parents routinely and effectively. This led to sensitization of the instrumental breadwinners of the family both outside the household through CHC efforts and within the household through their spouses. However, a traditional patriarchal culture of Bannu barred the involvement of women in our CHCs as women were not allowed to leave the house without the explicit permission of a male member of the family.

*“If the men were not at home, we had to approach that house the next day” (Businessperson, Stakeholder, Karachi).*

### Mechanisms for norm compliance

Throughout the project implementation phase, we found various occurrences where certain mechanisms were employed for norm compliance. These included social identity, power, the solving of social dilemmas, and rewards and punishments. Such mechanisms are not actively agreed upon but are implicitly ingrained in our social interactions as they provide a compulsive impetus to adhere to the norms of the social group.

*“[Stakeholder Name] helped us a lot as he has a good influence within the Sindhi community and he also asked political figures and polio workers to cooperate with us” (Project Staff, Karachi).*

While there were existing norms in place that fueled the refusal of the polio vaccine in our intervention communities, by leveraging these mechanisms we were able to create new social norms that led to a favorable opinion of the OPV, leading to a decrease in refusals within these communities. The core project strategy of conditional incentive delivery coupled with forming CHCs comprised of influential individuals in the community is where these mechanisms were their most apparent, something that we expand in our discussion below.

## Discussion

While our findings show the existence and acceptance of certain social norms regarding polio immunization in these communities within Karachi and Bannu, it is important to recognize the impact our intervention had through the establishment of CHCs and the provision of C3Is. The trial showed a significant decline in the refusals in our intervention UCs, while during the same time there was an increase in refusals for OPV in the control UCs, signifying the effectiveness of the intervention.

Multiple studies have shown the impact that CE has on boosting vaccine immunization rates (30, 69, 71–73). One intervention study conducted in Pakistan showed that by bundling polio vaccination with maternal and child healthcare through health camps leads to a significant uptake of all vaccines (30). Thus, their CE dealt more broadly with maternal and child health without a specific focus on polio immunization, arguing that vaccinating against polio as part of a health care package may be a more effective approach to involve religious leaders and local populations than polio-specific initiatives (30). However, such an approach fails to understand and change the existing social norms of a community regarding polio vaccinations and its refusals, specifically the administration of OPV through routine immunization campaigns (72). Thus, for our approach, we bundled the provision of incentives with the targets set for polio vaccination rates in our target communities. Studies that focused specifically on malaria vaccinations also had similar results through their interventions done for the uptake of the malaria vaccine, showing the power of CE for improved vaccination rates (73, 74). Furthermore, CE is effective when it extends beyond door-to-door engagement and community meetings (75), with having religious clerics and other influential community members serve as the promoters of the vaccine leading to a positive impact on vaccine acceptance (76).

In addressing refusals for polio vaccination, we recognized a common collective issue: individuals are often inclined to rely on the collective behavior of others for immunization coverage, a phenomenon identified as “free-riding” (61). Our intervention thus sought to address a critical question; why do individuals in the same community, subject to similar norms, exhibit drastically different personal beliefs regarding vaccination? Establishing conversations within communities, led by local representatives, became crucial. By having community members initiate and advocate for polio immunization, trust was fostered more effectively between vaccine-refusing households and CHCs (72, 77). However, initiating these conversations posed challenges (Table 2). Recruiting stakeholders was initially difficult due to the stigma surrounding polio in our target communities. The fluctuating schedule of polio campaigns further complicated trust-building, necessitating a new approach (Table 5) to reduce frustration and strengthen community confidence in polio workers (72).

**Table 5:** Successes of the project regarding polio immunization and beyond.

Successes of the project	Acceptance toward community participatory approach.
	Chronic refusals agreed to allow their children to be given the polio vaccine.
	Sustained participation in community engagement activities. Willingness to continue with polio awareness campaigns after the project period had ended.
	Betterment of community relationships. Increased trust, candor, and camaraderie between residents.
	Empowerment of the community through incentives, allowing them to better their economic conditions by allowing for income generation through sewing machines and vegetable carts, time saving through the provision of water pumps and solar panels in community spaces, and dignified mobility by providing wheelchairs and tricycles.

Our CHC-led dialogs, conducted in both public forums and private home visits, provided crucial flexibility. These settings allowed for confidential discussions during home visits and collective support in public forums, facilitating a comprehensive communication strategy (78). These dialogs served two essential purposes: first, to uncover core personal norms driving vaccine refusal, and second, to address these norms by dispelling misconceptions about the vaccine. Additionally, IEC materials underscored the benefits of vaccination and the risks of opting out, which helped parents better understand the implications of their decisions.

These public forums leveraged the social nature of individuals (79), relying on a norms-based approach that acknowledged the influence of societal expectations on behavior. The intervention recognized that personal beliefs are not shaped in isolation but are influenced by how individuals perceive their actions within their social context. Changing certain behaviors becomes an easier task then if it also focuses on empirical and normative expectations of an individual as opposed to solely driven toward changing their personal norms in a vacuum (61). Hence, observing the disapproval of vaccine refusals within the community was found to prompt parents to reconsider their views (80). A key mechanism of change was the implicit power of well-respected stakeholders, such as local school principals, Imams, and social activists, whose established trust within the community gave their opinions significant weight (71). This was in stark contrast to the coercive power that was created through the presence of police (one that parents and stakeholders alike disapproved of). Furthermore, this hierarchical structure of power was explicitly stated by one of our stakeholders. As school principals, they knew that their opinions mattered even outside the ambit of the school. Thus, while some teachers from the schools were also involved in the CHCs and in their role of teachers at the school engaged with parents in parent-teacher meetings (PTMs) or through home visits, the principals saw their awareness activities have the most impact on community parents.

Another central mechanism that emerged was one of social identity. While this somewhat overlapped with power, a religious and/or linguistic commonality (in other words, social identity) proved effective too. One stakeholder from Karachi pointed out how it became easier for him to assuage the concerns of Pakhtun parents as he spoke the same Pashto language as them, while some respondents from Bannu appreciated the inclusion of their Imam in the CHCs as it helped build trust especially when most of the misinformation surrounding polio vaccines comes from various religious figures through mass media sources.

The last mechanism seen in action was one that focused on solving social dilemmas through rewards and punishments (71). This was leveraged in the communications by CHC members who detailed the presence of polio in the community’s water samples and the importance of immunization. Yet solving these dilemmas required a significant uptake in polio vaccination rates – with their children facing the possibility of becoming crippled for life as a punishment. The reward came in the form of C3Is delivered to the communities that hit their target conversions from refusers to vaccine acceptors (and in some cases, promoters) (Table 5). These targets were reducing refusals by 30 and 50% in the two incentive delivery tranches, respectively, with all 11 clusters in Karachi and seven out of 10 clusters in Bannu successfully getting at least one incentive delivery over the course of this intervention. Crucially, the entire community collectively decided these incentives to provide maximum social benefit, sidestepping the need to make direct cash payments to each household.

Thus, these incentives proved crucial in our intervention because of the theoretical tipping point (61). Literature suggests that for a new social norm to become the prevalent norm, it must reach a tipping point, in other words reach critical mass (61, 81). This can happen well before the 51% simple majority as even before that there is enough adoption of this new norm (and a rejection of the old norm) that it becomes an empirical expectation for the community (61). However, this tipping point is dependent upon the ‘tightness–looseness’ found in our communities (61, 82) (Table 6). The ‘tightness–looseness’ theory posits that it is difficult to bring change in existing social norms in communities that already enjoy a high degree of social cohesion and vice versa (61). In Bannu, we found that due to the strong communal ties, if one household refused to participate in CHC activities or continued to refuse the OPV, the entire community would follow suit regardless of that household’s relative positionality in the hierarchical structure of the community. This was worsened by the culture of guns and violence at this site, evidenced by several terrorist attacks that took place during the project’s lifetime (83–86) (Table 2). Furthermore, a patriarchal norm of barring women from leaving their homes led to some women in our CHCs withdrawing from participating in its activities. Yet on the other end, the communities in Karachi were too loose. While that did provide an advantage of new norms finding the space to develop, a complete lack of communality hindered the initial effectiveness of our stakeholders, despite their relatively respected stature in their areas.

**Table 6:** Socio-demographic characteristics of study participants.

Indicators	*N* = 141
Age in years (mean SD)	39.00 ± (13.06)
Number of children	
1–2	58/132 (43.94%)
3–4	50/132 (37.88%)
5+	24/132 (18.18%)
Gender	
Men	70/141 (49.65%)
Women	71/141 (50.35%)
Level of education	
Illiterate/ uneducated	55/141 (39.00%)
Primary (<5 Years)	1/141 (0.70%)
Secondary (6–10 years)	34/141 (24.12%)
Intermediate (11–12 years)	22/141 (15.60%)
Graduate (13–14 years)	24/141 (17.03%)
Master (15–16 years)	4/141 (2.85%)
Other	1/141 (0.70%)
Occupation	
Housewife	62/139 (44.60%)
Managerial/Professional	26/139 (18.71%)
Skilled agricultural, forestry and fishery workers	23/139 (16.54%)
Service and sales workers	23/139 (16.54%)
Unemployed	5/139 (3.60%)
Language	
Balochi	1/89 (1.12%)
Gujarati	1/89 (1.12%)
Hindko	4/89 (4.49%)
Pushto	63/89 (70.78%)
Saraiki	1/89 (1.12%)
Sindhi	3/89 (3.37%)
Urdu	16/89 (17.98%)

To this end, the provision of community-driven incentives through our intervention, crucially in two tranches, built upon the CE activities and supercharged these empirical expectations toward the tipping point. By allowing the incentives to be decided by the community, and more importantly, actively include them during the delivery and deployment, a sense of ownership and personal investment was cultivated within the intervention communities. This resulted in greater involvement with the CE activities as community members actively recognized both the health and non-health benefits of polio immunization. Furthermore, delivering the incentives in two tranches led to increased trust in the intervention program and a shift in the optics surrounding polio immunization campaigns, resulting in an increased uptake of vaccines in the communities where the incentives were delivered. The shift in optics increased the goodwill between the project staff, the stakeholders, and the community. The knock-on effects of this were that there was a reported betterment in community relationships and a will for the community-based stakeholders to want to continue this work even after the project period ended. This marker of sustainability extended to the provided incentives whereby many respondents in both Karachi and Bannu expressed their willingness to maintain and perform any necessary repair work for the equipment they had received to ensure it remains functional and continues to benefit the community.

### Limitations and strengths

It is very difficult to preempt the ‘tipping point’ when concerned with the change in social norms, and computational models that have tried to predict this point rely on isolated factors without factoring in the social mechanisms driving the change. This *post hoc* awareness of the tipping point makes it difficult to refine future interventions where such a tipping point is reached quicker and more efficiently.

However, most research on social norms theory has been done either in laboratory conditions or is focused on alcohol consumption as opposed to vaccine uptake/immunization rates. While some research on this topic has utilized social norms theory, none have made use of both CE and C3Is to test the change in social norms. Furthermore, by harnessing and utilizing the implicit mechanisms for norm compliance within communities, our study gains a better understanding, as opposed to solely focusing on community engagement as a tool, of how a shift in the perception of vaccines, and subsequently the uptake of vaccines was brought about.

## Conclusion

This study shows that by combining community engagement with C3Is, changes in social norms can be made possible when concerned with childhood immunization. The subsequent increase in polio vaccine uptake can be used as a springboard for further behavioral changes within communities resistant to change and can serve as a helpful tool in the global fight against polio eradication especially after the recent surge in polio cases in Pakistan, we now know that the goal of polio eradication cannot be fulfilled with business-as-usual.

## Data Availability

The datasets presented in this article are not readily available because the data has personal identifiers of our study participants. Further inquiries can be directed to the corresponding author.
